# Energetic electron irradiations of amorphous and crystalline sulphur-bearing astrochemical ices

**DOI:** 10.3389/fchem.2022.1003163

**Published:** 2022-09-26

**Authors:** Duncan V. Mifsud, Péter Herczku, Richárd Rácz, K. K. Rahul, Sándor T. S. Kovács, Zoltán Juhász, Béla Sulik, Sándor Biri, Robert W. McCullough, Zuzana Kaňuchová, Sergio Ioppolo, Perry A. Hailey, Nigel J. Mason

**Affiliations:** ^1^ Centre for Astrophysics and Planetary Science, School of Physical Sciences, University of Kent, Canterbury, United Kingdom; ^2^ Institute for Nuclear Research (Atomki), Debrecen, Hungary; ^3^ Department of Physics and Astronomy, School of Mathematics and Physics, Queen’s University Belfast, Belfast, United Kingdom; ^4^ Astronomical Institute, Slovak Academy of Sciences, Tatranská Lomnica, Slovakia; ^5^ School of Electronic Engineering and Computer Science, Queen Mary University of London, London, United Kingdom

**Keywords:** astrochemistry, planetary science, electron irradiation, radiation chemistry, amorphous ice, crystalline ice, sulphur

## Abstract

Laboratory experiments have confirmed that the radiolytic decay rate of astrochemical ice analogues is dependent upon the solid phase of the target ice, with some crystalline molecular ices being more radio-resistant than their amorphous counterparts. The degree of radio-resistance exhibited by crystalline ice phases is dependent upon the nature, strength, and extent of the intermolecular interactions that characterise their solid structure. For example, it has been shown that crystalline CH_3_OH decays at a significantly slower rate when irradiated by 2 keV electrons at 20 K than does the amorphous phase due to the stabilising effect imparted by the presence of an extensive array of strong hydrogen bonds. These results have important consequences for the astrochemistry of interstellar ices and outer Solar System bodies, as they imply that the chemical products arising from the irradiation of amorphous ices (which may include prebiotic molecules relevant to biology) should be more abundant than those arising from similar irradiations of crystalline phases. In this present study, we have extended our work on this subject by performing comparative energetic electron irradiations of the amorphous and crystalline phases of the sulphur-bearing molecules H_2_S and SO_2_ at 20 K. We have found evidence for phase-dependent chemistry in both these species, with the radiation-induced exponential decay of amorphous H_2_S being more rapid than that of the crystalline phase, similar to the effect that has been previously observed for CH_3_OH. For SO_2_, two fluence regimes are apparent: a low-fluence regime in which the crystalline ice exhibits a rapid exponential decay while the amorphous ice possibly resists decay, and a high-fluence regime in which both phases undergo slow exponential-like decays. We have discussed our results in the contexts of interstellar and Solar System ice astrochemistry and the formation of sulphur allotropes and residues in these settings.

## Introduction

It has been established for some time now that the laboratory irradiation of astrochemical ice analogues using energetic charged particles (i.e., ions and electrons) or ultraviolet photons may lead to the production of prebiotic molecules relevant to biology, such as amino acids or nucleobases (e.g., Muñoz-Caro et al., 2002; Hudson et al., 2008; Nuevo et al., 2012). Motivated by a desire to further understand the non-equilibrium chemistry leading to the formation of these so-called ‘seeds of life’, many studies have sought to determine and quantify the influence of various physical parameters on the outcome of such reactions. Perhaps the best studied of these is ice temperature, with previous works having demonstrated the key influence of this parameter on the abundance of product molecules formed after irradiation (e.g., Sivaraman et al., 2007; Mifsud et al., 2022a).

Our recent work has also demonstrated that the solid phase of an irradiated ice plays a crucial role in determining the outcome of astrochemical reactions mediated by ionising radiation. Through a series of comparative electron irradiations, we have demonstrated that the radiolytic decay rate of an astrochemical ice is dependent upon the nature, strength, and extent of the intermolecular interactions that characterise its solid phase (Mifsud et al., 2022b; Mifsud et al., 2022c). For instance, the decay rate of α-crystalline CH_3_OH was found to be significantly less rapid than that of the amorphous phase. This was attributed to the existence of an extensive network of strong hydrogen bonds that exists in the α-crystalline phase. This network requires an additional energy input from the projectile electrons to be overcome, thus leaving less energy overall to drive radiolytic chemistry. Conversely, the amorphous CH_3_OH ice is characterised only by localised clusters of hydrogen bonded molecules. Such a structure does not benefit from the same stabilising effect supplied by the network of hydrogen bonds in the α-crystalline phase, particularly as hydrogen bonding in CH_3_OH is known to be a cooperative phenomenon in which the presence of one hydrogen bond in the network strengthens successive hydrogen bonds through electrostatic effects (Kleeberg and Luck 1989; Sum and Sandler 2000).

In the case of N_2_O ice, the decay rate of the amorphous phase was noted to be only moderately more rapid than that of the crystalline phase (Mifsud et al., 2022b). The dominant intermolecular forces of attraction in solid N_2_O are expected to be dipole-dipole interactions. Although the orientation of these dipoles in the crystalline phase is anticipated to confer some degree of resistance against radiolytic decay compared to the amorphous phase, this is considerably less than that induced by the hydrogen bonding network in α-crystalline CH_3_OH. This therefore explains the more similar radiolytic decay rates of amorphous and crystalline N_2_O. Such results carry important implications for the radiation processing of astrochemical ices, as they suggest that the irradiation of amorphous ices is more chemically productive than that of crystalline ones; particularly in the case of those ices which are able to form strong and extensive intermolecular bonds when crystalline. Extending this idea further, it is entirely possible that those astrophysical environments in which space radiation-induced amorphisation processes dominate over thermally-induced crystallisation may be characterised by a more productive radiation chemistry. This idea is not unreasonable, particularly in light of the discovery of several complex organic molecules in pre-stellar cores (e.g., McGuire et al., 2020; Burkhardt et al., 2021).

In this present study, we have expounded upon our previous work by performing comparative electron irradiations of the crystalline and amorphous phases of pure H_2_S and SO_2_ astrochemical ice analogues, thus simulating the processing such ices undergo during their interaction with galactic cosmic rays, stellar winds, or magnetospheric plasmas as a result of the production of large quantities of secondary electrons (Mason et al., 2014; Boyer et al., 2016). Solid H_2_S is known to exhibit a number of stable crystalline phases under low temperature and ambient pressure conditions (Fathe et al., 2006), but it is the crystalline phase III (hereafter simply referred to as the crystalline H_2_S phase) which is of importance under conditions relevant to astrochemistry. This phase is orthorhombic, having eight molecules per unit cell and adopting the *Pbcm* space group. SO_2_ may also exist as an orthorhombic crystalline solid under astrochemical conditions, but in this case the *Aba*2 space group is adopted and there are only two molecules per unit cell (Schriver-Mazzuoli et al., 2003).

Although sulphur is one of the most abundant elements in the cosmos and is of importance in both biochemical and geochemical contexts, much remains unknown regarding its chemistry in interstellar and outer Solar System settings (Mifsud et al., 2021a). It is thought, for instance, that H_2_S ice processing by galactic cosmic rays or ultraviolet photons accounts for the apparent depletion of sulphur (relative to its total cosmic abundance) in dense interstellar clouds by producing large quantities of atomic sulphur or molecular sulphur chains and rings which are difficult to detect using current observation techniques (Jiménez-Escobar and Muñoz-Caro 2011; Jiménez-Escobar et al., 2014). H_2_S itself has not yet been definitively detected in interstellar icy grain mantles (Boogert et al., 2015). Conversely, SO_2_ ice has been detected within both the dense interstellar medium as well as on the surfaces of outer Solar System bodies such as the Galilean moons of Jupiter (Boogert et al., 1997; Carlson et al., 1999). However, the exact chemical mechanisms leading to its formation in these settings remain widely debated (Mifsud et al., 2021a).

The purpose of this study is thus two-fold: (i) to determine whether the phase of irradiated sulphur-bearing molecular ices influences the radiation-induced rate of decay as was previously demonstrated for non-sulphur-bearing ices; and (ii) to contribute further to our (comparatively poor) understanding of the extra-terrestrial chemistry of sulphur. To achieve these goals, the amorphous and crystalline phases of pure H_2_S and SO_2_ ices were respectively irradiated with 2 and 1.5 keV electrons, and the resultant physico-chemical changes were followed *in situ* using Fourier-transform mid-infrared (FT-IR) transmission absorption spectroscopy.

## Experimental methodology

**FIGURE 1:**
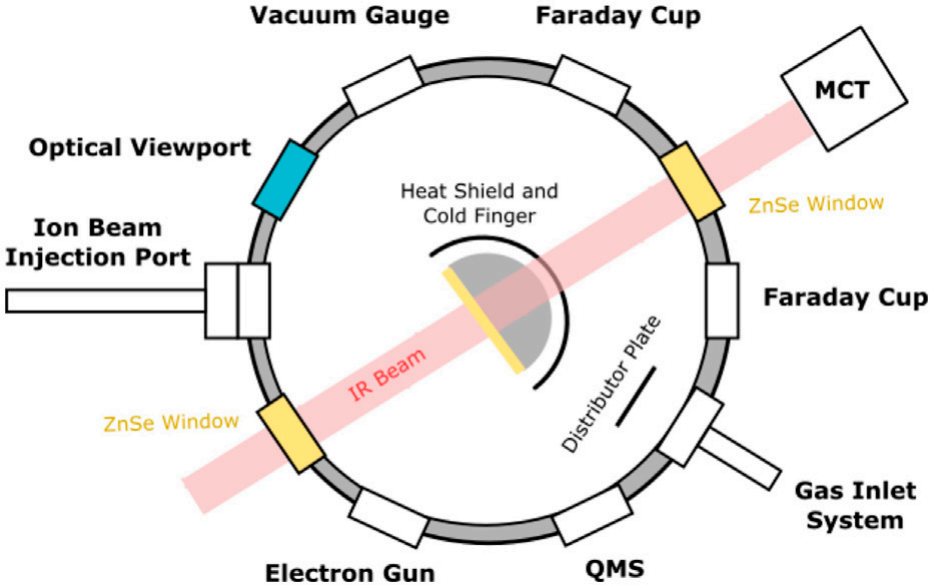
Top-view schematic diagram of the ICA set-up. Note that electron irradiations are carried out such that projectile electrons impact the target ices at 36° to the normal. Figure reproduced from Mifsud et al. (2021b) with the kind permission of the European Physical Journal (EPJ).

The irradiation experiments were performed using the Ice Chamber for Astrophysics-Astrochemistry (ICA); a custom-built experimental apparatus located at the Institute for Nuclear Research (Atomki) in Debrecen, Hungary. This apparatus (Figure 1) has been described in detail in previous publications (Mifsud et al., 2021b; Herczku et al., 2021), and so only a brief description of the most salient details will be provided here. The ICA is a UHV-compatible chamber with a nominal base pressure of a few 10^−9^ mbar which is achieved by the combined action of a dry rough vacuum pump and a turbomolecular pump. Within the centre of the chamber is a gold-coated oxygen-free copper sample holder which supports up to four ZnSe deposition substrates, onto which astrochemical ice analogues may be prepared. The temperature of the sample holder and the substrates may be cooled to 20 K using a closed-cycle helium cryostat, although an operational temperature range of 20–300 K is available.

The preparation of H_2_S and SO_2_ astrochemical ice analogue phases was achieved via background deposition by allowing the relevant gas into a pre-mixing line before dosing it into the main chamber at a pressure of a few 10^−6^ mbar. Amorphous ice phases were prepared by deposition at 20 K, while crystalline H_2_S and SO_2_ ices were prepared by deposition at 60 and 90 K, respectively, before being cooled to 20 K. Once deposited, FT-IR spectra (spectral range = 4,000–650 cm^−1^; spectral resolution = 1 cm^−1^) of the ices were acquired, from which quantitative measurements of their molecular column densities *N* (molecules cm^−2^) and thicknesses *d* (μm) could be performed by measuring the peak area *P* (cm^−1^) of a characteristic absorption band (Eq. 1):

$$d=10,000\times \frac{NZ}{N_{A}\rho }=10,000\times \ln\left(10\right)\times \frac{PZ}{A_{\nu }N_{A}\rho }$$ (1)

where *Z* is the molar mass (g mol^−1^) of the molecular ice, *N*_A_ is the Avogadro constant (6.02×10^23^ molecules mol^−1^), *ρ* is the density of the ice (g cm^−3^), and *A*_ν_ is the band strength constant of the characteristic absorption band whose area is being measured (cm molecule^−1^). Information on the molecular column densities and thicknesses of the ices investigated in this study, as well as the physical parameters used to calculate these values, is given in Tables 1 and 2.

**TABLE 1:** List of physical parameters and constants used for the quantitative study of the deposited H_2_S and SO_2_ astrochemical ices.

Physical parameter	H_2_S	SO_2_	References
Absorption Band Position (cm^−1^)	2,550	1,148	Garozzo et al. (2008) and Hudson and Gerakines (2018)
Amorphous *A*_ν_ (10^−17^ cm molecule^−1^)	1.12	0.22	Garozzo et al. (2008) and Hudson and Gerakines (2018)
Crystalline *A*_ν_ (10^−17^ cm molecule^−1^)	2.90	0.88	Garozzo et al. (2008) and Hudson and Gerakines (2018)
Amorphous *T*_deposition_ (K)	20	20	This work
Crystalline *T*_deposition_ (K)	60	90	This work
*T*_irradiation_ (K)	20	20	This work
*Z* (g mol^−1^)	34	64	This work
Density (g cm^−3^)	1.22	1.89	Post et al. (1952) and Yarnall and Hudson (2022)
*E*_electron_ (keV)	2.0	1.5	This work
Maximum Electron Penetration Depth (nm)	155	70	Drouin et al. (2007)

**TABLE 2:** List of initial molecular column densities and thicknesses of the H_2_S and SO_2_ ices investigated in this study.

Ice	Species	Phase	*N* (10^17^ molecules cm^−2^)	*d* (μm)
1	H_2_S	Amorphous	7.05	0.326
2	H_2_S	Amorphous	6.50	0.301
3	H_2_S	Amorphous	7.67	0.355
*Average*			*7.07*	*0.327*
4	H_2_S	Crystalline	5.78	0.268
5	H_2_S	Crystalline	6.35	0.294
6	H_2_S	Crystalline	7.61	0.352
*Average*			*6.58*	*0.305*
7	SO_2_	Amorphous	3.09	0.174
8	SO_2_	Amorphous	2.49	0.140
9	SO_2_	Amorphous	2.89	0.162
*Average*			*2.82*	*0.159*
10	SO_2_	Crystalline	2.62	0.147
11	SO_2_	Crystalline	2.19	0.123
12	SO_2_	Crystalline	2.84	0.160
*Average*			*2.55*	*0.143*

The deposited pure H_2_S and SO_2_ astrochemical ices were respectively irradiated using 2 and 1.5 keV electron beams (average flux = 4×10^13^ electrons cm^−2^ s^−1^) to a total fluence of about 8.3×10^16^ electrons cm^−2^, with projectile electrons impacting the target ices at an angle of 36° to the normal. Prior to commencing the irradiations, the beam current, spot size, and profile homogeneity were determined using the method described by Mifsud et al. (2021b). CASINO simulations (Drouin et al., 2007) of the trajectory of the electrons as they travelled through the solid ices revealed that the maximum penetration depths of the incident electrons into the H_2_S and SO_2_ ices were 155 and 70 nm, respectively. FT-IR spectra were collected at several intervals throughout the irradiation process so as to monitor the radiation chemistry occurring. All irradiations were carried out at 20 K so as to preclude any temperature-dependent effects on the mobility of radiolytically derived radicals. Moreover, the irradiation of each ice phase was performed three times so as to ensure good repeatability of the experiment.

## Results and discussion

**FIGURE 2:**
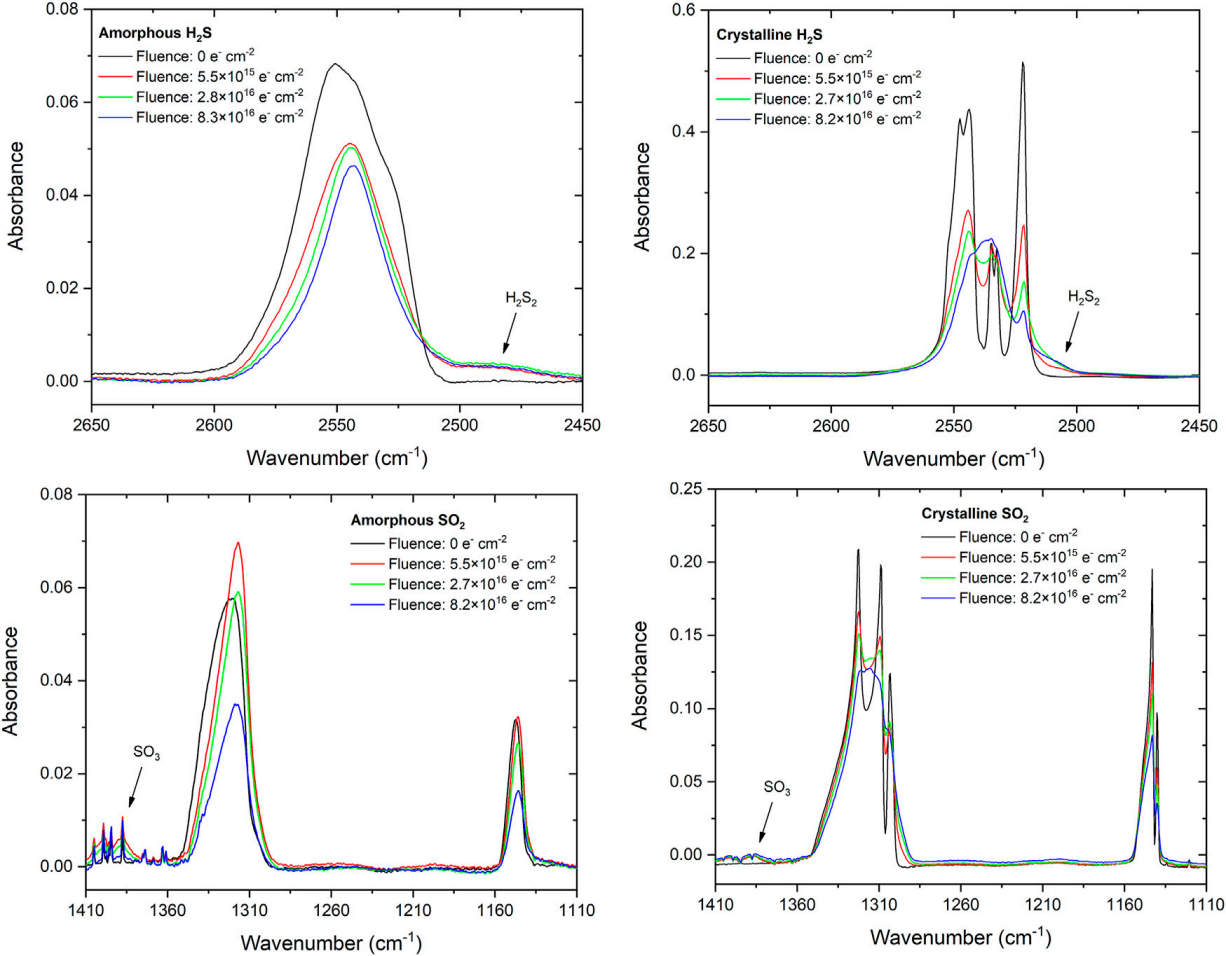
FT-IR spectra of the amorphous and crystalline phases of H_2_S and SO_2_ ices at several points during their irradiation by energetic electrons at 20 K. Note that the fine structures coincident with the SO_3_ absorption band in the spectrum of the electron irradiated amorphous SO_2_ ice are caused by instabilities in the purge of the detector. Moreover, the initial increase in the intensity of the amorphous SO_2_ asymmetric stretching mode is likely caused by the radiation-induced compaction of the porous ice.

The FT-IR spectra of the pure H_2_S and SO_2_ ice phases investigated in this study, both before and after irradiation by electrons at different fluences, are depicted in Figure 2. In the amorphous phase, H_2_S presents a very broad absorption band which peaks at 2,550 cm^−1^ attributable to both the symmetric (*ν*_1_) and asymmetric (*ν*_3_) stretching modes. In the crystalline phase, this band is better resolved and the individual contributors may be observed. The asymmetric stretching mode is observed to peak at 2,546 cm^−1^, while the symmetric stretching mode is split into two components peaking at 2,534 and 2,522 cm^−1^. Fathe et al. (2006) also observed this splitting and attributed it to the existence of two unique sulphur atoms and three unique S–H bonds in the unit cell.

The amorphous phase of solid SO_2_ presents two distinct albeit broad absorption asymmetric and symmetric stretching mode bands which respectively peak at 1,320 and 1,148 cm^−1^. In the crystalline phase, these bands are observed to be better resolved, with three and two individual structures being observed in the asymmetric and symmetric stretching mode bands, respectively. These structures have been attributed to the various isotopologues of SO_2_ (Schriver-Mazzuoli et al., 2003): the bands peaking at 1,323, 1,309, 1,303, and the shoulder band at 1,301 cm^−1^ are ascribed to the transverse B_1_(TO) and B_2_(TO) modes of ^32^S^16^O_2_, and to ^34^S^16^O_2_ and ^32^S^16^O^18^O; while those peaking at 1,143 and 1,140 cm^−1^ are respectively attributed to the transverse A_1_(TO) mode of ^32^S^16^O_2_ and to ^34^S^16^O_2_. It is interesting to note that the naturally low abundances of ^34^S and ^18^O do not result in weaker band intensities for the SO_2_ isotopologues containing these isotopes due to intermolecular coupling between these isotopologues in the condensed phase (Brooker and Chen 1991; Schriver-Mazzuoli et al., 2003).

The onset of electron irradiation brings about noticeable changes in the appearances of the spectra of the pristine ices. Perhaps the most prominent of these is the significant broadening of the crystalline ice absorption bands, which also lose their resolved individual structures. This is due to radiation-induced amorphisation, which has been well documented in several ice species irradiated by ions, electrons, and ultraviolet photons; including H_2_O, CH_3_OH, N_2_O, and NH_3_ (e.g., Kouchi and Kuroda 1990; Moore et al., 2007a; Famá et al., 2010; Mifsud et al., 2022b; Mifsud et al., 2022c). It is interesting to note that, even at the end of the irradiation process once a fluence of >8×10^16^ electrons cm^−2^ has been delivered to the crystalline ices, the appearances of their absorption bands are still not identical to those of the deposited amorphous ices. Indeed, small signs of crystallinity (e.g., the presence of shoulder bands or shifted band peak positions) are still observable in the crystalline ices at the end of irradiation. As such, these irradiated ices are likely largely amorphous but with some small degree of remnant structural order.

The irradiation of molecular ices is known to initiate a rich chemistry leading to the formation of new species. Previous studies have established that irradiated H_2_S ices efficiently yield H_2_S_2_ as well as higher order polysulphanes (H_2_S_*x*_, where *x* > 2) in addition to allotropic forms of elemental sulphur (Shingledecker et al., 2020; Cazaux et al., 2022). In our experiments, we have observed the formation of H_2_S_2_ through the development of its vibrational stretching modes which appear as a broad shoulder band on the lower wavenumber end of the analogous H_2_S absorption bands at about 2,500 cm^−1^ (Figure 2; Moore et al., 2007b). The chemistry leading to the formation of H_2_S_2_ (as well as higher order polysulphanes) is thought to be largely mediated by HS radicals formed via the dissociation of the parent H_2_S molecules:

$$H_{2}S\;\to HS+H$$ (2)

$$2HS\to H_{2}S_{2}$$ (3)

It should be noted that HS radicals produced as a result of the radiolytic dissociation of H_2_S may pick up an electron to form HS^−^ions. These HS^−^ions may possibly participate in chemistry leading to the formation of other HS radicals via proton abstraction reactions with H_2_S, after which the resultant HS^−^ion may undergo electron auto-detachment to yield HS. A similar process has recently been demonstrated to occur in H_2_O ice with respect to radiolytically derived OH radicals and OH^−^ions (Kitajima et al., 2021).

The irradiation of the SO_2_ ice phases was also observed to lead to the formation of new molecules; in particular SO_3_ which was observed through its asymmetric stretching absorption band at 1,388 cm^−1^ (Guldan et al., 1995). SO_3_ formation in irradiated SO_2_ ices has been studied extensively and is believed to be the result of the dissociation of the latter species to yield free oxygen atoms which may then bond with other SO_2_ molecules (Moore et al., 2007b). It should be noted, however, that earlier studies by Pilling and Bergantini (2015) and de Souza Bonfim et al. (2017) have demonstrated that electronically excited SO_2_ may also abstract oxygen atoms from either an adjacent SO_2_ molecule or from a O_2_ molecule; the latter having likely been formed as a result of the double ionisation of the SO_2_ parent molecule followed by electron neutralisation as described recently by Wallner et al. (2022):

$${SO}_{2}\to SO+O$$ (4)

$${SO}_{2}+O\to {SO}_{3}$$ (5)

$${SO}_{2}^{*}+{SO}_{2}\left(or\;O_{2}\right)\to {SO}_{3}+SO\left(or\;O\right)$$ (6)

**FIGURE 3:**
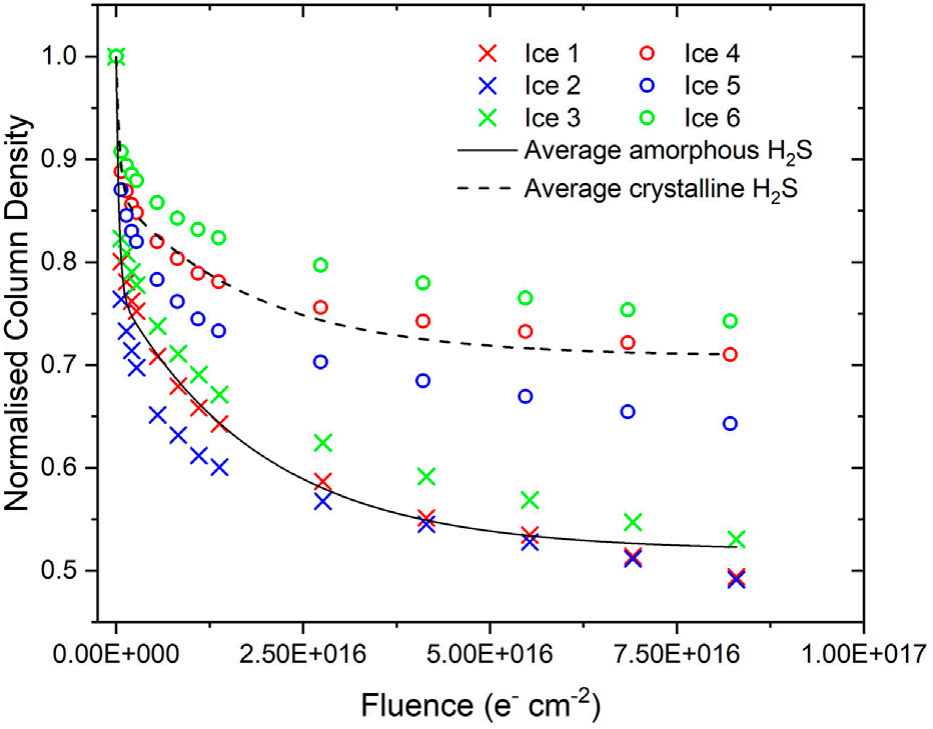
Decay of amorphous and crystalline H_2_S column densities normalised to the initially deposited column density during irradiation using 2 keV electrons. Note that the average decay trends are fitted by two exponential decay functions joined at a fluence of 1.4 × 10^15^ electrons cm^−2^.

Differences in the parent molecule decay trends and in the abundance of molecular products observed after irradiation were noted between the studied amorphous and crystalline ice phases. Considering first the decay trends of the amorphous and crystalline H_2_S ices: it was noted that the rate of decay of the crystalline phase was significantly slower than that of the amorphous phase (Figure 3). A similar trend was observed during the comparative electron irradiations of the amorphous and crystalline phases of CH_3_OH, N_2_O, and H_2_O ices (Mifsud et al., 2022b; Mifsud et al., 2022c). This was attributed to the additional energy input required to disrupt the extensive intermolecular forces of attraction that characterise the crystalline solid before radiolytic chemistry as a result of molecular dissociation may proceed. In CH_3_OH, the α-crystalline phase contains extensive arrays of cooperative and strong hydrogen bonds which stabilise the ice considerably against radiolytic decay compared to the amorphous solid, which is only characterised by localised hydrogen bonds (Kleeberg and Luck 1989; Sum and Sandler 2000; Mifsud et al., 2022b).

H_2_S is also capable of forming hydrogen bonds between adjacent molecules (Das et al., 2018), although these are significantly weaker than those in alcohols: the hydrogen bond strengths in pure CH_3_OH and H_2_S are 6.3 and 1.0 kcal mol^−1^, respectively (Pellegrini et al., 1973; Bhattacherjee et al., 2013). Despite this weaker nature of the hydrogen bond in H_2_S, it still displays a relative stability of the crystalline phase to radiation-induced decay (compared to the amorphous phase) that is qualitatively similar to that of CH_3_OH. As such, it is likely that another factor should be invoked to account for the relative radio-resistance of the crystalline phases of these ices; that of lattice energies. The energetic advantage induced by the ordering of the molecular components of the solid ice must also be overcome and thus a proportion of the incident electrons’ kinetic energy must be expended upon overcoming both the lattice energy as well as the more extensive hydrogen bonding network in the crystalline phase, leaving less energy to induce the molecular dissociation that drives radiolytic chemistry.

Such an interpretation is wholly consistent with our previously reported results on the comparative electron irradiations of amorphous and crystalline N_2_O, which demonstrated only a moderately more rapid decay rate of the former compared to the latter (Mifsud et al., 2022b). In that case, a fraction of the kinetic energy of the incident electrons must be used to overcome both the increased ordering of the molecular dipoles as well as the crystal lattice energy. We also note that *A*_ν_ differs by a factor-of-two-and-a-half between the amorphous and crystalline H_2_S phases, with that of the latter being greater. This is not insignificant, and the rapid amorphisation of the crystalline phase as a result of its irradiation may mean that column density measurements of this phase may be somewhat underestimated and, as such, the radio-resistance of the crystalline phase may be even greater than that depicted in Figure 3, although this is difficult to quantify.

**FIGURE 4:**
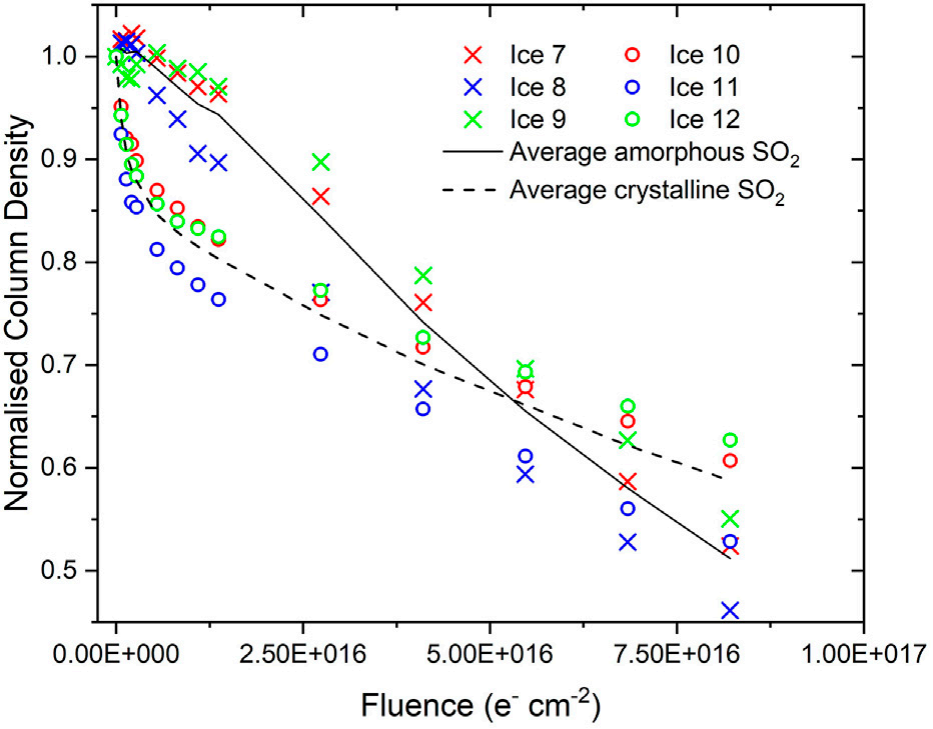
Decay of amorphous and crystalline SO_2_ column densities normalised to the initially deposited column density during irradiation using 1.5 keV electrons. Note that the average decay trends are not fits and are plotted solely to guide the eye.

The radiation-induced decay trends of amorphous and crystalline SO_2_ (Figure 4), however, are significantly different to those of H_2_S and the previously studied ices. The decay trend of the crystalline SO_2_ ice initially exhibits the anticipated profile of a rapid exponential decay. However, once a fluence of about 1.4×10^16^ electrons cm^−2^ is exceeded, the normalised column density declines significantly more slowly. Perhaps even more surprising is the fact that the amorphous SO_2_ normalised column density (with respect to the initial SO_2_ column density deposited) does not really vary at low electron fluences, having an average normalised column density of 0.97 after a fluence of 8.2×10^15^ electrons cm^−2^ had been delivered. For comparison, by the point this fluence had been delivered to the crystalline SO_2_ ice, its average normalised column density had decreased to 0.83. However, similarly to the case of the crystalline SO_2_ ice, once a fluence of about 1.4×10^16^ electrons cm^−2^ had been delivered, the normalised column density was observed to undergo a slow exponential-like decay. Interestingly, beyond a delivered fluence of 1.4×10^16^ electrons cm^−2^, the rate of decay of the amorphous SO_2_ is greater than that of the crystalline SO_2_, and indeed the average decay trends cross one another at a fluence of about 5.3×10^16^ electrons cm^−2^.

Providing an exact reason for the observed amorphous SO_2_ decay trends is a challenging task. Measurements of the photo-desorption of SO_2_ molecules from an amorphous ice induced by soft x-rays allowed de Souza Bonfim et al. (2017) to suggest that, at low fluences, the recombination of fragments produced by the dissociation of SO_2_ to yield electronically excited SO_2_ may be a favourable process, thus largely precluding net SO_2_ dissociation within the ice. It is also possible that the irradiation of the amorphous SO_2_ ice results in its compaction, which may cause an increase in *A*_ν_ of the measured band (Figure 2). Similar results were recently shown for ion irradiated amorphous CO ice, for which *A*_ν_ very rapidly increased by about 5% of its nominal value as a result of the compaction of the ice (Ivlev et al., 2022). It is not possible to discount either of these possible explanations based on the available evidence.

**FIGURE 5:**
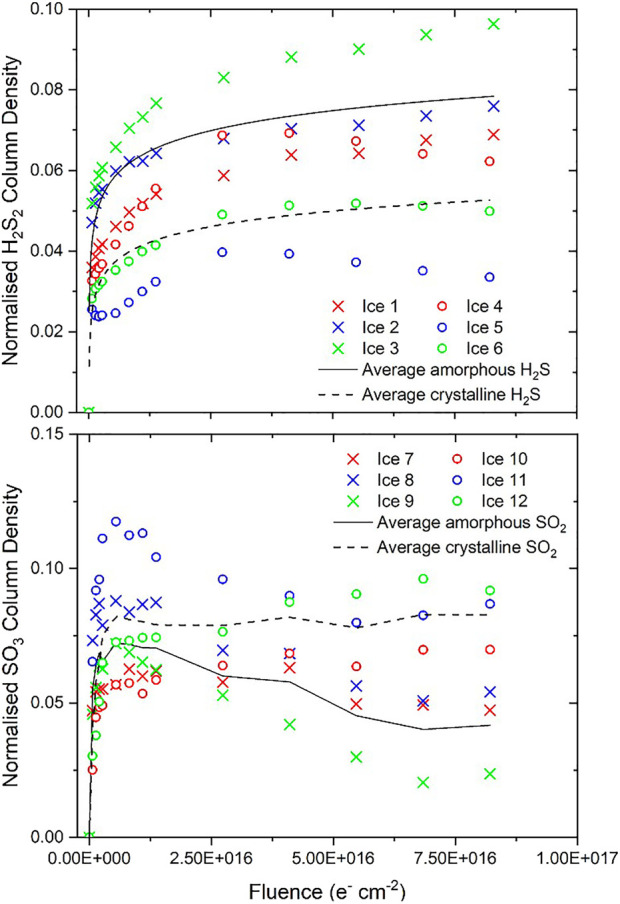
*Above:* Column density of H_2_S_2_ from amorphous and crystalline H_2_S ices irradiated using 2 keV electrons at 20 K. *Below:* Column density of SO_3_ from amorphous and crystalline SO_2_ ices irradiated using 1.5 keV electrons at 20 K. Column densities have been normalised to the initially deposited column density of the parent molecular ice. Note that in the case of H_2_S_2_ the average trends are fitted by logarithmic functions while in the case of SO_3_ the average trends are not fits and are plotted solely to guide the eye.

As a final analytical consideration, we have attempted to establish the sulphur budget of the electron irradiation processes presented in this study. The possible chemical transformations of H_2_S and SO_2_ to infrared inactive atomic or allotropic forms of sulphur have already been referred to (Shingledecker et al., 2020; Cazaux et al., 2022), and so it is useful to quantify how much of the initially deposited H_2_S or SO_2_ ice ends up in such a form as a result of its irradiation. As depicted in Figure 2, the only major infrared active products of H_2_S and SO_2_ irradiation were H_2_S_2_ and SO_3_, respectively. We have quantified the column densities of these product molecules throughout the irradiation processes by measuring the peak areas of their primary absorption bands and making use of Eq. (1) (Figure 5). We note that we have taken *A*_ν_ for the H_2_S_2_ absorption band at about 2,500 cm^−1^ to be 2.4×10^−17^ cm molecule^−1^ (Cazaux et al., 2022). To the best of our knowledge, *A*_ν_ has not yet been defined for the SO_3_ absorption band at 1,388 cm^−1^, and so we have followed the example of de Souza Bonfim et al. (2017) who assumed that this band strength is equal to that of the SO_2_ asymmetric stretching mode which is 1.47×10^−17^ cm molecule^−1^ (Garozzo et al., 2008).

As expected, the yield of H_2_S_2_ from the irradiated amorphous H_2_S ice is greater than that from the irradiated crystalline H_2_S ice, commensurate with the increased decay rate of the former compared to the latter. Conversely, the electron irradiation of the crystalline SO_2_ ice proved to be more conducive to the formation of SO_3_ than did the irradiation of the amorphous phase. This is as expected for the low-fluence regime of the irradiation process (up to a fluence of about 5.3×10^16^ electrons cm^−2^), due to the amorphous SO_2_ ice possibly resisting radiolytic decay. However, the greater abundance of SO_3_ in the irradiated crystalline ices persists even beyond this fluence, despite the more rapid decay of amorphous SO_2_ after this point. It should be noted, however, that after peaking at a fluence of about 5.5×10^15^ electrons cm^−2^, the SO_3_ column density within the irradiated amorphous SO_2_ ice also declines slightly (Figure 5). The concomitant loss of SO_2_ and SO_3_ from the ice during its irradiation suggests that sulphur is either being converted into a form which is not infrared active (e.g., atomic or allotropic sulphur) or is being desorbed or sputtered from the bulk ice. In either case, however, there is a fraction of the initially deposited sulphur that remains unobserved in the ice.

**FIGURE 6:**
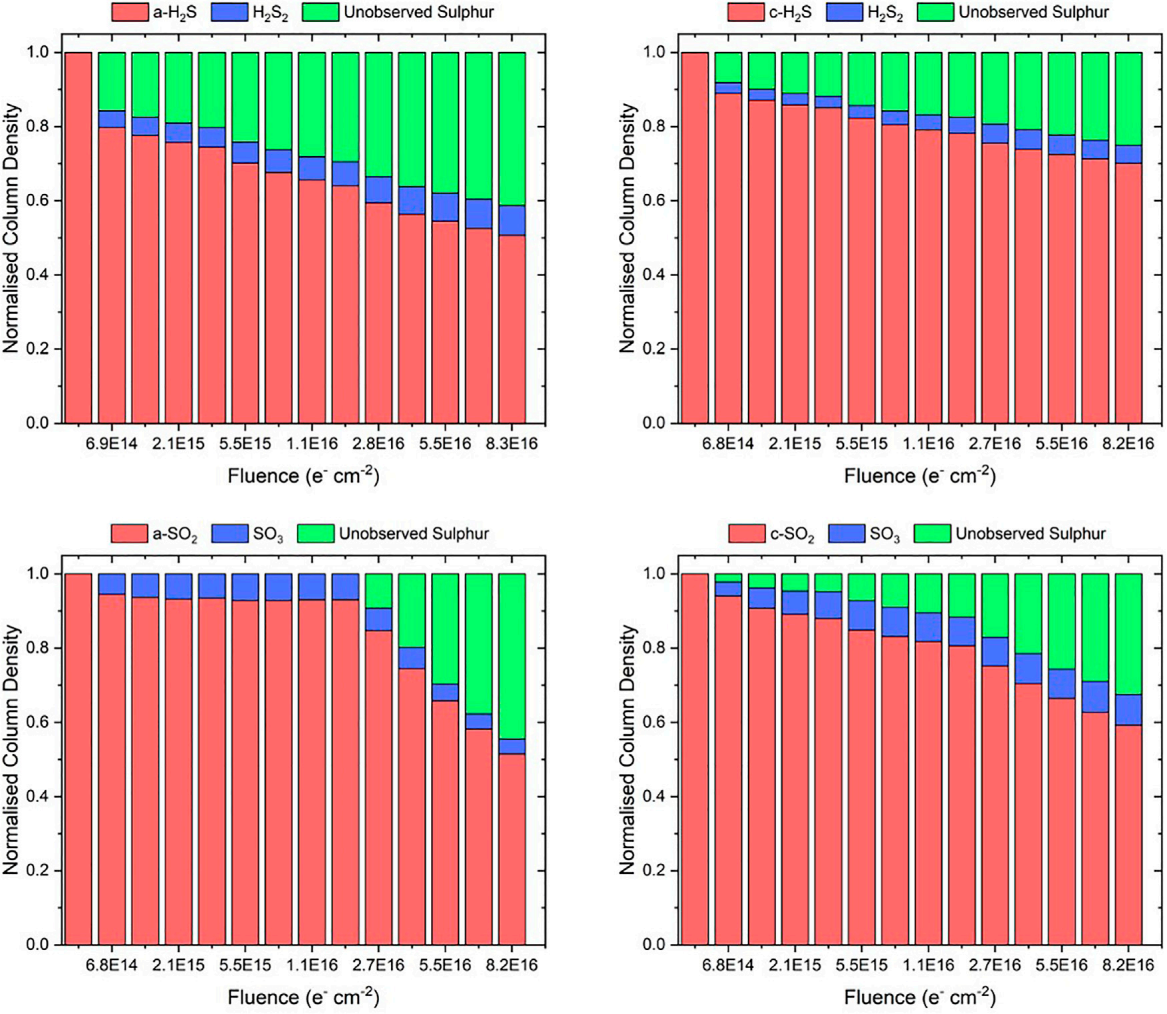
Sulphur budgets of the electron irradiated amorphous and crystalline H_2_S and SO_2_ ices considered in this study. Uncertainties in the normalised abundance of the parent and primary product molecules are estimated to be within 3%. The quantity of unobserved sulphur represents an upper bound for the abundance of atomic or allotropic sulphur formed as a result of irradiation, since it is not known how many (if any) sulphur-containing species were sputtered or desorbed from the bulk ice. Note that the notations “a-” and “c-” used in the caption indicate whether the irradiated ice is amorphous or crystalline.

The sulphur budgets of each of the irradiated ices considered in this study are shown in Figure 6. It is possible to note that a loss of sulphur is observed upon supplying an initial electron fluence of 6.9×10^14^ electrons cm^−2^ in all of the ices apart from the amorphous SO_2_ ice, and that the quantity of unobserved sulphur as a fraction of that initially deposited continually grows during irradiation. In the case of the amorphous SO_2_ ice, unaccounted for sulphur is only registered after a fluence of 2.7×10^16^ electrons cm^−2^ has been supplied, possibly due to the resistance of SO_2_ to radiolytic dissociation as discussed earlier (de Souza Bonfim et al., 2017).

Although it is possible that electron irradiation resulted in the sputtering or desorption of the parent ice species, we consider this process to have likely been a relatively minor one. Previous work has shown, for example, that the reactive desorption of H_2_S upon its formation as a result of the hydrogenation of HS on the surface of an interstellar ice analogue has a probability of 3% per hydrogenation event (Oba et al., 2018; Oba et al., 2019; Furuya et al., 2022), which is small from the perspective of an experimental study. Thus, if the electron-induced sputtering or desorption of sulphur-bearing molecules from the bulk ice is assumed to be negligible, then the fractions of unobserved sulphur shown in Figure 6 represent the sulphur present in an infrared inactive form, such as atomic sulphur or, more likely, residues composed of sulphur allotropes (Gomis and Strazzulla 2008). Our data therefore suggest an important point with regards to the production of such residues from pure H_2_S and SO_2_ ices: it is apparent that the irradiation of amorphous ices results in a greater abundance of sulphur residues than does the irradiation of crystalline ices. It should be noted, however, that the conversion of observable molecular sulphur to unobservable residues is very efficient in each of the considered ices, with amorphous H_2_S, crystalline H_2_S, amorphous SO_2_, and crystalline SO_2_ ices respectively showing 41, 25, 44, and 32% conversion of the initially deposited sulphur to residues at the end of irradiation (Figure 6).

## Implications for interstellar and Solar System chemistry

The results of this present study are directly applicable to the astrophysical chemistry of sulphur. In dense molecular clouds in the interstellar medium, there is a known paucity of observed sulphur relative to its expected cosmic abundance (Tieftrunk et al., 1994; Ruffle et al., 1999). Recent studies have suggested that this depletion may be mediated by the Coulomb-enhanced freeze-out of sulphur cations onto negatively charged dust grains, whereupon they polymerise to yield sulphur-bearing residues and chains (Cazaux et al., 2022). Modelling efforts have suggested different explanations as to the major forms of sulphur in interstellar space: Vidal et al. (2017) suggested that, depending upon the age of the dense cloud, the majority of the sulphur exists either as unobservable atoms in the gas phase or as H_2_S within icy grain mantles. Navarro-Almaida et al. (2020) also suggested the dominance of gas-phase sulphur atoms, whilst Laas and Caselli (2019) concluded that the majority of sulphur is found as organosulphur molecules within the icy grain mantles. Shingledecker et al. (2020) proposed that the major sulphur-bearing species in the condensed phase were sulphur allotropes along with SO_2_ and OCS.

Nonetheless, it is expected that H_2_S will be present within icy grain mantles as a result of the hydrogenation of adsorbed sulphur atoms. Furthermore, SO_2_ is suspected to be present in such ices on the basis of its tentative detection (Boogert et al., 1997). Our results demonstrate that the irradiation of these ices by galactic cosmic rays (for which we have used an energetic electron beam as a simulant) could further contribute to the presence of sulphur residues, chains, and atoms in the dense interstellar medium and, by extension, could also account for a portion of the depleted sulphur in such regions. Moreover, such processes are likely to be more efficient when the dense cloud is either fairly young (i.e., it is still in its pre-stellar stage) or in those regions of the cloud which are not in proximity to heat sources such as proto-stars since, under such conditions, the interstellar icy grain mantles would not undergo crystallisation or thermal segregation of their molecular constituents and would thus remain amorphous.

Our results are also applicable to outer Solar System chemistry, particularly in the cases of the Galilean moons of Jupiter and of comets. SO_2_ is the dominant molecular component of the surface ices and exosphere of the innermost of the Galilean moons; Io (Douté et al., 2001), and has also been detected as a component of the icy surfaces of Europa, Ganymede, and Callisto (Noll et al., 1997; Domingue et al., 1998; Noll et al., 1998). Surface temperatures on Io undergo quotidian cycles between 90–130 K, thus allowing for cycles of sublimation and condensation of the surface SO_2_ frosts to be maintained (Bagenal and Dols 2020). During the Ionian day, warmer temperatures cause the sublimation of much of the surface SO_2_ ice, resulting in the formation of a tenuous exosphere. At night, however, lower temperatures drive the collapse of much of the exosphere and re-condensation of the SO_2_ to surface ices.

Given that Io orbits within the giant Jovian magnetosphere, its surface is continually exposed to ionising radiation in the form of energetic ions and electrons. The flux of 0.1–52 keV electrons at the surface of Io was given by Frank and Paterson (1999) to be 3.1×10^8^ electrons cm^−2^ s^−1^, meaning the fluence delivered in our experiments would be delivered to the Ionian surface within 8.5 years. The temperature conditions at the surface of Io would lead one to assume that SO_2_ ice is naturally found in the crystalline phase, and that, therefore, the radiation-induced formation of SO_3_ should be reasonably efficient (Figure 5). However, our results also demonstrate that the prolonged irradiation of crystalline SO_2_ ice at 20 K results in its amorphisation, reducing the comparative yield of SO_3_ in favour of refractory residues of allotropic sulphur (Figure 6). Such residues may contribute to the distinct colouration of Io (Carlson et al., 2007). It should be noted, however, that the extrapolation of radiation-induced amorphisation results acquired at low temperatures to higher ones may not be appropriate. For instance, although the amorphisation of crystalline H_2_O is known to occur efficiently as a result of its irradiation at 20 K, this process has never been reported at temperatures >70 K (Mifsud et al., 2022c). The efficiency of the radiation-induced crystalline SO_2_ ice amorphisation process at various temperatures (including those relevant to the surface of Io) should therefore be tested in future experiments.

Finally, we note that our results are also applicable to the chemistry occurring within the icy nuclei of comets. The recent ESA *Rosetta* mission to comet 67P/Churyumov-Gerasimenko revealed the presence of a number of sulphur-bearing molecules within its icy nucleus, including H_2_S, SO_2_, SO, OCS, CS_2_, and S_2_ (Rubin et al., 2020). As the comet approaches perihelion in its orbit around the Sun, thermally-induced crystallisation processes begin to out-compete space radiation-induced amorphisation. Correspondingly, the formation of allotropic sulphur residues via the irradiation of the H_2_S and SO_2_ cometary ice components by the solar wind may decrease slightly in line with the results presented in Figure 6.

## Conclusion

In this experimental study, we have performed comparative and systematic electron irradiations of the amorphous and crystalline phases of H_2_S and SO_2_ ices using 2 and 1.5 keV electrons, respectively. We have shown that, in the case of H_2_S, the amorphous parent ice decays at a more rapid rate than does the crystalline one, in a manner that is similar to that previously reported for CH_3_OH (Mifsud et al., 2022b). This has been attributed to the presence of a more structured and extensive hydrogen bonding system in the crystalline phase compared to the amorphous phase, as well as the inherent lattice energy of the former, which require an additional energy input from the projectile electrons to be overcome before radiolytic chemistry may proceed. The formation of H_2_S_2_ as a product of the electron irradiation of H_2_S occurs to a greater extent in the amorphous phase than in the crystalline phase, in part due to the greater abundance of radiolytically generated HS radicals.

The irradiation of the SO_2_ ice revealed unexpected results. In the amorphous ice, two regimes are apparent: a low-fluence regime in which the ice is possibly resistant to radiolytic decay (potentially due to the favourable reformation of excited SO_2_ after the dissociation of ground-state SO_2_) and a high-fluence regime in which a slow exponential-like decay trend is observed. This contrasts greatly with the crystalline ice, for which a rapid exponential decay is first observed in the low-fluence regime followed by a slower decay (which is slower than that of the amorphous phase) in the high-fluence regime. Interestingly, the formation of SO_3_ as a result of the irradiation of the crystalline ice was always greater than during the irradiation of the amorphous ice, possibly due to the initial resistance of the amorphous ice to radiolytic decay and its subsequent preferential formation of infrared inactive sulphur allotropes and residues.

We suggest that our results are important not only in the context of further investigating the phase-dependent radiation chemistry of astrochemical ices, which has thus far been overlooked in the literature, but also in further understanding the chemistry of sulphur in extra-terrestrial environments. Our characterisation of this phase-dependent chemistry is directly applicable to understanding the sulphur chemistry on the surface of Io, as well as in the icy nuclei of comets. Moreover, our calculated sulphur budgets for each of the irradiation processes considered in this study (which reveal the seemingly efficient formation of infrared inactive sulphur allotropes and residues) may aid in further constraining the exact molecular forms of sulphur in interstellar icy grain mantles and cometary ices. Finally, we conclude by noting that our experimental results further demonstrate the importance of incorporating ice phase as a factor when designing more complete ‘systems astrochemistry’ investigations (Mason et al., 2021).

## Data Availability

The raw data supporting the conclusion of this article will be made available by the authors, without undue reservation.
